# Inflammatory biomarkers may be associated with poor outcomes after mechanical thrombectomy

**DOI:** 10.1186/s12959-024-00630-7

**Published:** 2024-07-09

**Authors:** Hong Wang, Xiaobing Tian, Zhangyuan Liao, Xuanye Yue, Libin Sun, Xingrong Li, Ming Zou, Jiayue Ding

**Affiliations:** Department of Neurology, Tianjin Medical University General Hospital, Tianjin, 300052 China

**Keywords:** Acute stroke, Endovascular treatment, Inflammation, Ischemic stroke

## Abstract

**Background:**

Mechanical thrombectomy (MT) has become the mainstay of treatment for acute ischemic stroke (AIS) recently. This case-control study aimed to identify the pivotal role of inflammation in the prognosis of AIS patients after MT.

**Methods:**

Altogether, 70 AIS patients who underwent MT were retrospectively recruited for this study. Receiver operating characteristic analysis was performed to demonstrate the sensitivity and specificity of the inflammatory variables for predicting prognosis. A meta-analysis was performed to pool the published results together. Stata software was used for analysis.

**Results:**

There was no differences in pre-MT inflammatory biomarkers between patients who survived and those who died, as well as patients with modified Rankin Scale (mRS) 0–2 and mRS ≥ 3. In contrast, post-MT C-reactive protein (CRP) levels might be a potential parameter to predict death after thrombectomy [area under the curve (AUC), 95%confidence interval (CI), 0.737, 0.587–0.887; *p* = 0.005; optimal cutoff value = 4.565]. Moreover, post-MT monocyte count might be an appropriate parameter to predict poor long-term prognosis after thrombectomy (AUC, 95%CI, 0.704, 0.575–0.833; *p* = 0.017; optimal cutoff value = 0.345). A meta-analysis revealed that the pre-MT inflammatory indices, including white blood cell count (weighted mean difference, 95%CI, 1.32, 1.01—1.63), neutrophil count (1.23, 0.95—1.51), monocyte count (0.05, 0.02—0.09), neuthrophil-to-lymphocyte ratio (2.42, 1.98—2.87) and platelet-to-lymphocyte ratio (24.65, 7.99—41.32), were higher in patients with 3-month mRS ≥ 3, and the lymphocyte count (−0.31,−0.43 to −0.18) was lower in this cohort.

**Conclusions:**

Inflammatory indices were significantly associated with the prognosis of patients undergoing MT, especially post-MT CRP and monocyte count, which can predict long-term outcomes.

**Supplementary Information:**

The online version contains supplementary material available at 10.1186/s12959-024-00630-7.

## Background

Mechanical thrombectomy (MT) has become the mainstay of treatment for acute ischemic stroke (AIS) in recent years [1]. MT can achieve a successful recanalization rate of 41–88%, which is much higher than that yielded by traditional therapies, including intravenous thrombolysis [2]. However, the functional outcome is limited, with only 30–50% of patients who achieve successful recanalization after MT having a good prognosis [3]. In theory, the early restoration of blood flow can improve neurological disorders caused by ischemia. The mismatch between the rate of successful reperfusion and good prognosis is associated with various factors in which the inflammatory response plays an essential role [4]. It is well-known that a substantial inflammatory response occurs immediately after brain infarction, including neutrophil infiltration, lymphocyte redistribution, monocyte invasion, and proinflammatory cytokine release [5]. Several inflammatory indices, especially adopted blood sample indices such as leukocyte, neutrophil, lymphocyte, and monocyte counts, are widely used to evaluate the severity and predict the prognosis of stroke in clinical settings [6]. These inflammatory indices are also available for AIS patients undergoing MT. The inflammatory status before MT may influence brain injury after reperfusion, and the inflammatory status after MT appears to affect brain repair in the long run [7]. Therefore, both baseline and post-MT inflammatory indices are considered to be linked to the efficacy of recanalization; this has garnered the attention of researchers in recent years owing to its practical value because it appears to be a safe, low-cost, and easy-to-use strategy for predicting the prognosis of patients undergoing MT [4]. However, these studies yielded conflicting results and did not provide any convincing conclusions [7]. In this regard, we analyzed the cohort in our hospital and conducted a meta-analysis to identify the pivotal role of inflammation in the prognosis of AIS patients after MT.

## Methods

### Study population

Altogether, 70 AIS patients who underwent MT at Tianjin Medical University General Hospital from January 2020 to September 2022 were retrospectively recruited for this study (Figure S1). The informed consent for undergoing MT had been obtained from all subjects. There were no age or sex restrictions. Some patients underwent intravenous thrombolysis before MT. This was a retrospective case-control study which only recruits the patients screened by the inclusion/exclusion criteria. The inclusion criteria were as follows: (1) AIS patients with a National Institutes of Health Stroke Scale (NIHSS) score ≥ 6 [8]; (2) onset-to-puncture time (OPT) ≤ 6 h, or 6–24 h with mismatch volume > 15 ml as assessed by computed tomography perfusion imaging (according to Dawn Trial); (3) Alberta Stroke Program Early CT Score (ASPECTS) ≥ 6; (4) the thrombectomy sites included internal carotid artery (ICA), middle cerebral artery (MCA), basilar artery (BA), and vertebral artery (VA); and (5) the occluded vessels achieved successful recanalization (TICI 2b or 3) after thrombectomy. Exclusion criteria were as follows: (1) ischemic events caused by stent or carotid endarterectomy; (2) patients comorbid with infectious diseases (such as pneumonia, urinary infection and septicopyemia), autoimmune diseases, cancer, trauma and other complications that might influence the body immune system within 72 h after MT; (3) deaths or poor outcomes caused by cardiac diseases, digestive system diseases, and/or other medical complications; and (4) participants lacking complete data.

According to the Cochrane systematic review and the Preferred Reporting Items, a meta-analysis was performed in this study. PubMed, Embase and ClinicalTrials.gov were searched for publications written by English related to specific keywords, such as ‘thrombectomy,’ ‘inflammation,’ and ‘immunology,’ which were published prior to March 2023 (A detailed search strategy was shown in Table S1). The references of the retrieved articles were thoroughly reviewed subsequently for additional reports that might have been missed in our search. The enrolled studies fulfilled the following criteria: (1) patients treated with MT alone or MT plus thrombolysis; (2) case-control analysis for comparisons between 3-month modified Rankin Scale (mRS) ≤ 2 and mRS > 2 [9], and/or survivors and deaths in the long run; and (3) inflammatory markers presented as means ± standard deviation (SD) or median (interquartile range, IQR). Exclusion criteria were as follows: (1) cohorts comprising patients treated with thrombolysis alone; (2) inflammatory markers presented with odds ratio (OR) and relative risk (RR); and (3) end-point events including hemorrhage, edema, early neurological deterioration, and short-term mRS.

### Data collection

Clinical data included demographics, OPT, risk factors, baseline stroke severity, clinical outcomes, and inflammatory biomarkers (the type of apparatus was XS800, sysmex and Backman image-800), such as white blood cells (WBC), neutrophils, lymphocytes, monocytes, C-reactive protein (CRP), and hyper-sensitive C-reaction protein (hs-CRP). The neutrophil-to-lymphocyte ratio (NLR) indicated the ratio of neutrophil count to lymphocyte count; the lymphocyte-to-monocyte ratio (LMR) indicated the ratio of lymphocyte count to monocyte count; the platelet-to-lymphocyte ratio (PLR) indicated the ratio of platelet count to lymphocyte count; and the systemic immune-inflammation index (SII) was indicated by platelet×neutrophil/lymphocyte. In addition, the severity and prognosis of stroke were evaluated using NIHSS and mRS scores at admission and follow-up.

### Statistical analysis

Stata 15.1 was used for the analysis in this study. Continuous variables following a Gaussian distribution were expressed as mean ± SD and analyzed with Student’s t-test; otherwise, the data were presented as median (IQR) and analyzed using the Mann–Whitney U test. Categorical variables are presented as numbers (percentages) and were analyzed using the Pearson χ2 test or Fisher’s exact test. Furthermore, a logistic regression model was used to adjust confounding effects (including age, OPT, NIHSS at admission, thrombectomy site, pre-MT ASPECTS, all of them which were considered to be correlated with the safety and efficacy of MT). The associations between inflammatory biomarkers and baseline NIHSS were assessed using Pearson correlation. The associations between inflammatory biomarkers and clinical outcomes were assessed using Spearman’s correlation. Receiver operating characteristic (ROC) analysis was performed to demonstrate the sensitivity and specificity of the variables for predicting prognosis. The area under the ROC curve (AUC) reflected the accuracy of prediction, and the power was further evaluated with positive predictive value (+ PV), negative predictive value (-PV), positive likelihood ratio (+ LR), and negative likelihood ratio (-LR). Optimal cutoff values were considered along with maximal Youden’s index [10]. Statistical significance was set at *p* < 0.05.

As for meta-analysis, available data was processed using Stata software (version 15.0 SE) in this study. Only the mean (SD) was used for the pooled analysis. A funnel plot was used to evaluate publication bias (Using RevMan 5.3 software). Chi-Square test was used to assess the heterogeneity of data. Pooled analysis was conducted with the fixed-effects model using Mantel-Haenszel method when the heterogeneity was expected to be available (I^2^ < 50%). Otherwise, the random-effects model computed by the DerSimonian-Laird method was conducted (I^2^≥50%). Hypothesis testing was done using the U test expressed by Z and *P*-value. *P*-value < 0.05 were considered statistically significant.

## Results

### Population characteristics

A total of 70 patients were enrolled (52 males and 18 females, average age was 63.94 ± 11.59 years), of whom 87.1% had hypertension, 64.3% had diabetes, and 95.7% had hyperlipidemia. Thrombectomy sites included the ICA (31.4%), MCA (42.9%), BA (24.3%), and VA (1.4%). The average hospitalization time was 14.5 ± 4.3 days and the average follow-up time was 47.5 ± 23.9 days. The mean OPT was 6.48 ± 3.12 h and the baseline mean NIHSS score was 16.81 ± 7.25. The median (IQR) of pre-MT ASPECTS and post-MT ASPECTS were 8.0 (9.0, 10) and 6.0 (3.0, 8.0) respectively. A total of 23 patients (32.9%) died following MT, of whom 18 died during hospitalization (the average onset-to-dead time was 10.5 ± 4.3 days) and 5 during follow-up (the average onset-to-dead time was 45.4 ± 21.3 days). Patients with an mRS score 0–2 accounted for 7.1% of the patients at discharge and 21.4% at follow-up, while those with mRS 3–5 accounted for 67.1% of those at discharge and 45.7% at follow-up. The details aforementioned could be seen in Table 1. The differences of baseline characteristics between favorite and unfavorite outcomes (survivors versus deaths, and patients with follow-up mRS 0–2 versus mRS ≥ 3) were shown in Table S2 and Table S3.

**Table 1:** Baseline characteristics of stroke patients treated with MT

Characteristics		
Demographics	Number of patients (*n* = 70)	%
Male	52	74.3
Age, years	63.94 ± 11.59	-
History		
Hypertension	45	64.3
Diabetes	18	25.7
Atrial fibrillation	22	31.4
Brain infarction	15	21.4
Smoke	31	44.3
Drink	26	37.1
Thrombectomy site		
Internal carotid artery	22	31.4
Middle cerebral artery	30	42.9
Basal artery	17	24.3
Vertebral artery	1	1.4
Death		
During hospital	18	25.7
At follow-up time	5	7.1
mRS 0–2		
During hospital	5	7.1
At follow-up time	15	21.4
mRS 3–5		
During hospital	47	67.1
At follow-up time	32	45.7
Clinical presentations		
OPT, hours	6.49 ± 3.15	-
NIHSS score	16.81 ± 7.30	-
Follow-up time, days	47.5 ± 23.9	-
Pre-MT ASPECTS	9.0 (8.0, 10)	-
Post-MT ASPECTS	6.0 (3.0, 8.0)	-

mRS: modified Rankin Scale; OPT: onset-to-puncture time; NIHSS: National Institutes of Health Stroke Scale; ASPECTS: Alberta Stroke Program Early CT Score; MT: mechanism thrombectomy; -: data not available

### Association between pre-MT inflammatory parameters and prognosis

Fifty-four patients completed serum biomarker testing before MT. Pearson correlation analysis showed no correlation between the pre-MT parameters and baseline NIHSS scores (Table S4). Spearman’s correlation analysis showed that pre-MT monocyte counts (*r* = 0.314, *p* = 0.021) were correlated with follow-up mRS scores, despite a very low correlation (Table S5).

Sixteen patients (29.6%) died after MT, and this study found no differences in pre-MT inflammatory biomarkers between patients who survived and those who died (all *p*-value and adjusted *p*-value > 0.05). Parameters (WBC counts and monocyte counts) that had between-group differences with *p*-value < 0.1 were included in the ROC analysis. The AUC of WBC counts was 0.660, with 95% confidence interval (CI) 0.493–0.826 (*p* = 0.066), and that of monocyte counts was 0.661, with 95% CI 0.512–0.811 (*p* = 0.063). These results indicate that the pre-MT parameters in this study were not associated with death after MT (Table 2 and Table S6).

**Table 2:** Differences of pre-MT biomarkers between survivors and deaths after MT

Biomarkers	Survivors (38 cases)	Deaths (16 cases)	*p*-value	Adjusted *p*-value
WBC counts, 10^9^/L	7.75[6.77, 9.88]	9.41[7.22, 13.32]	0.066	0.243
Neutrophil counts, 10^9^/L	5.75[4.20, 7.53]	6.49[5.02, 11.93]	0.179	0.389
Lymphocyte counts, 10^9^/L	1.47 ± 0.69	1.56 ± 0.75	0.677	0.310
Monocyte counts, 10^9^/L	0.42[0.33, 0.64]	0.50[0.45, 0.70]	0.063	0.194
Neutrophil percentage, %	73.52 ± 11.99	75.34 ± 12.61	0.619	0.582
Lymphocyte percentage, %	19.12 ± 9.68	17.14 ± 10.10	0.501	0.674
Monocyte percentage, %	5.68 ± 2.48	5.68 ± 1.95	0.997	0.739
Eosinophils percentage, %	1.32 ± 1.11	1.51 ± 1.62	0.624	0.297
Basophils percentage, %	0.30[0.20, 0.43]	0.25[0.20, 0.38]	0.430	0.519
NLR	3.88[2.35, 6.81]	4.61[2.40, 11.50]	0.483	0.765
PLR	166.91[98.34, 221.97]	135.96[123.92, 180.69]	0.820	0.269
LMR	3.52 ± 1.56	2.94 ± 1.54	0.217	0.669
SII	760.21[463.14, 1521.24]	975.05[568.75, 2279.45]	0.483	0.658

MT: mechanism thrombectomy; WBC: white blood cell; NLR: neutrophil-to-lymphocyte ratio: indicating the ratio of neutrophil count to lymphocyte count; LMR: lymphocyte-to-monocyte ratio: indicating the ratio of lymphocyte count to monocyte count; PLR: the platelet-to-lymphocyte ratio: indicating the ratio of platelet count to lymphocyte count; and SII: systemic immune-inflammation index: indicating platelet×neutrophil/lymphocyte; *Adjusted for age: OPT: NIHSS at admission, thrombectomy site, pre-MT ASPECTS

Forty-four patients (81.5%) had mRS ≥ 3 at follow-up. Univariate analysis presented significant differences in pre-MT monocyte percentage (follow-up mRS 0–2 vs. mRS ≥ 3, 4.31 ± 2.46 vs. 5.99 ± 2.19, *p* = 0.037) between patients with mRS 0–2 and mRS ≥ 3 at follow-up, while the multivariate analysis did not find the differences between the two arms. Based on comparisons between patients with follow-up mRS 0–2 and mRS ≥ 3, the parameters (WBC counts and monocyte counts) that had between-group differences with *p*-value < 0.1 were included in the ROC analysis. The AUC of WBC counts was 0.400, with 95% CI 0.221–0.579 (*p* = 0.327), and that of monocyte counts was 0.689, with 95% CI 0.504–0.874 (*p* = 0.065). The results revealed that the pre-MT parameters in this study might be underpowered to predict the prognosis (Table 3 and Table S7).

**Table 3:** Differences of pre-MT biomarkers between patients with the follow-up mRS 0–2 and ≥ 3 after MT

Biomarkers	mRS 0–2 (10 cases)	mRS ≥ 3 (44 cases)	*p*-value	Adjusted *p*-value
WBC counts, 10^9^/L	8.74[7.41, 12.56]	8.19[6.48, 9.99]	0.066	0.239
Neutrophil counts, 10^9^/L	7.28[5.01, 10.52]	5.92[4.26, 7.95]	0.179	0.205
Lymphocyte counts, 10^9^/L	1.40 ± 0.51	1.52 ± 0.74	0.644	0.738
Monocyte counts, 10^9^/L	0.38[0.19, 0.52]	0.47[0.38, 0.69]	0.063	0.223
Neutrophil percentage, %	78.07 ± 12.65	73.15 ± 11.92	0.249	0.447
Lymphocyte percentage, %	16.20 ± 9.30	19.06 ± 9.87	0.408	0.507
Monocyte percentage, %	4.31 ± 2.46	5.99 ± 2.19	0.037	0.295
Eosinophils percentage, %	0.80[0.10, 2.23]	1.10[0.23, 2.40]	0.909	0.912
Basophils percentage, %	0.30[0.10, 0.43]	0.30[0.20, 0.40]	0.430	0.538
NLR	5.00[2.98, 11.74]	3.79[2.39, 7.04]	0.483	0.881
PLR	166.91[102.58, 283.96]	141.31[102.75, 212.78]	0.820	0.685
LMR	3.97 ± 0.95	3.20 ± 1.64	0.163	0.273
SII	1102.90[566.00, 3689.14]	730.87[513.70, 1478.06]	0.483	0.839

mRS: modified Rankin Scale; MT: mechanism thrombectomy; WBC: white blood cell; NLR: neutrophil-to-lymphocyte ratio: indicating the ratio of neutrophil count to lymphocyte count; LMR: lymphocyte-to-monocyte ratio: indicating the ratio of lymphocyte count to monocyte count; PLR: the platelet-to-lymphocyte ratio: indicating the ratio of platelet count to lymphocyte count; and SII: systemic immune-inflammation index: indicating platelet×neutrophil/lymphocyte; *Adjusted for age, OPT, NIHSS at admission, thrombectomy site, pre-MT ASPECTS

### Association between post-MT inflammatory parameters and prognosis

A total of 67 patients completed serum biomarker testing after MT. The average post-MT serum collection time was 1.8 ± 0.9 days. Spearman’s correlation analysis showed that post-MT lymphocyte percentage (*r*=−0.287, *p* = 0.019), monocyte counts (*r* = 0.266, *p* = 0.030), NLR (*r* = 0.272, *p* = 0.026) and LMR (*r*=−0.317, *p* = 0.009) were correlated with follow-up mRS scores, despite a very low correlation (Table S8).

Twenty-three patients (34.3%) died after the MT. Univariate analysis showed significant differences in post-MT LMR (survivors vs. death, 2.57[1.29, 5.89] vs. 1.53[1.07, 3.55], *p* = 0.025) and CRP levels (2.24[0.90, 6.36] vs. 9.68[4.09, 14.90], *p* = 0.005) between patients who survived and those who died, while multivariate analysis indicated that post-MT WBC counts (survivors vs. death, OR, 95%CI, 0.806, 0.785–0.827, adjusted *p* = 0.043), CRP levels (0.824, 0.803–0.845, adjusted *p* = 0.008) and hs-CRP levels (0.968, 0.943–0.993, adjusted *p* = 0.032) were statistically different between patients who survived and those who died. ROC analysis was performed with the post-MT parameters that had between-group differences with a *p*-value < 0.1 (including WBC counts, monocyte counts, lymphocyte counts, NLR, LMR, SII, CRP levels, hs-CRP levels). The parameters with AUC > 0.7 included post-MT CRP levels (AUC, 95%CI, 0.737, 0.587–0.887, *p* = 0.005) and hs-CRP levels (0.710, 0.501–0.920, *p* = 0.061); however, only CRP levels reached statistical significance. Moreover, the parameter with AUC > 0.7 was further calculated with an optimal cutoff value to predict death after thrombectomy. The optimal cutoff value for CRP levels was 4.565 (+ PV = 0.609, -PV = 0.857, +LR = 2.850, -LR = 0.305). The patients were classified into two groups according to the optimal cutoff values to validate the predictive power of post-MT CRP levels for death after thrombectomy. Four out of 28 patients with post-MT CRP < 4.565 and 14 out of 23 with CRP ≥ 4.565 died after thrombectomy (*p* = 0.001, kappa value was 0.475). The results demonstrated that post-MT CRP levels might be a potential parameter to predict death after thrombectomy (Table 4 and Table S6).

**Table 4:** Differences of post-MT biomarkers between survivors and deaths after MT

Biomarkers	Survivors (44 cases)	Deaths (23 cases)	*p*-value	Adjusted *p*-value
WBC counts, 10^9^/L	10.59 ± 3.40	12.08 ± 2.94	0.080	0.043
Neutrophil counts, 10^9^/L	9.14 ± 3.31	10.45 ± 2.60	0.105	0.057
Lymphocyte counts, 10^9^/L	0.96[0.63, 1.21]	0.83[0.48, 1.06]	0.210	0.654
Monocyte counts, 10^9^/L	0.25[0.13, 0.69]	0.58[0.35, 0.88]	0.060	0.127
Neutrophil percentage, %	87.75[89.15, 90.88]	89.00[83.40, 92.50]	0.335	0.443
Lymphocyte percentage, %	8.65[6.03, 12.80]	6.30[4.40, 9.70]	0.055	0.349
Monocyte percentage, %	3.00[1.35, 7.08]	5.00[2.90, 7.70]	0.300	0.536
Eosinophils percentage, %	0.00[0.00, 0.20]	0.00[0.00, 0.10]	0.492	0.736
Basophils percentage, %	0.15[0.10, 0.20]	0.10[0.10, 0.20]	0.679	0.779
NLR	9.86[6.25, 14.45]	14.11[8.40, 21.63]	0.068	0.081
PLR	228.24[162.39, 303.75]	257.14[178.16, 495.56]	0.496	0.123
LMR	2.57[1.29, 5.89]	1.53[1.07, 3.55]	0.025	0.073
SII	2069.38[1057.21, 3102.88]	2826.12[1764.56, 4629.16]	0.091	0.064
CRP, mg/dL	2.24[0.90, 6.36]	9.68[4.09, 14.90]	0.005	0.008
hs-CRP, mg/L	14.44[6.72, 35.26]	44.81[13.88, 101.43]	0.062	0.032

MT: mechanism thrombectomy; WBC: white blood cell; NLR: neutrophil-to-lymphocyte ratio: indicating the ratio of neutrophil count to lymphocyte count; LMR: lymphocyte-to-monocyte ratio: indicating the ratio of lymphocyte count to monocyte count; PLR: the platelet-to-lymphocyte ratio: indicating the ratio of platelet count to lymphocyte count; and SII: systemic immune-inflammation index: indicating platelet×neutrophil/lymphocyte; CRP: C-reactive protein; hs-CRP: hyper-sensitive C-reaction protein; *Adjusted for age, OPT, NIHSS at admission, thrombectomy site, pre-MT ASPECTS

After MT, 52 patients (77.6%) had mRS score ≥ 3 at follow-up. Univariate analysis showed that post-MT monocyte counts (follow-up mRS 0–2 vs. mRS ≥ 3, 0.20[0.12, 0.34] vs. 0.49[0.17, 0.93], *p* = 0.017), monocyte percentage (2.00[1.30, 3.60] vs. 5.90[1.63, 7.68], *p* = 0.032) and LMR (5.04[2.21, 5.90] vs. 1.60[1.07, 4.37], *p* = 0.008) were significantly different between patients with mRS 0–2 and mRS ≥ 3 at follow-up, while the multivariate analysis did not find the differences between the two cohorts. The post-MT monocyte counts, monocyte percentage, and LMR had between-group differences with *p*-value < 0.1, and were therefore further analyzed using ROC analysis. The parameter with AUC > 0.7 only included post-MT monocyte counts (AUC, 95%CI, 0.704, 0.575–0.833, *p* = 0.017), and it was further calculated with optimal cutoff value to predict mRS ≥ 3 at 3 months. The optimal cutoff value of monocyte counts was 0.345 (+ PV = 0.919, -PV = 0.400, +LR = 3.270, -LR = 0.433). Altogether, 18 of 30 patients with post-MT monocyte counts < 0.345 and 34 of 37 with monocyte counts ≥ 0.345 were confirmed as mRS ≥ 3 at follow-up after MT (*p* = 0.002, kappa value was 0.335). The results suggest that post-MT monocyte counts might be an appropriate parameter to predict poor long-term prognosis after thrombectomy (Table 5 and Table S7).

**Table 5:** Differences of post-MT biomarkers between patients with the follow-up mRS 0–2 and ≥ 3 after MT

Biomarkers	mRS 0–2 (15 cases)	mRS ≥ 3 (52 cases)	*p*-value	Adjusted *p*-value
WBC counts, 10^9^/L	10.39 ± 3.59	11.31 ± 3.23	0.348	0.150
Neutrophil counts, 10^9^/L	9.11 ± 3.66	9.73 ± 2.99	0.498	0.196
Lymphocyte counts, 10^9^/L	0.94[0.75, 1.19]	0.89[0.57, 1.19]	0.573	0.904
Monocyte counts, 10^9^/L	0.20[0.12, 0.34]	0.49[0.17, 0.93]	0.017	0.051
Neutrophil percentage, %	88.20[80.40, 90.60]	87.95[81.30, 91.35]	0.816	0.936
Lymphocyte percentage, %	10.00[6.40, 13.80]	7.60[4.83, 10.60]	0.121	0.707
Monocyte percentage, %	2.00[1.30, 3.60]	5.90[1.63, 7.68]	0.032	0.067
Eosinophils percentage, %	0.00[0.00, 0.60]	0.00[0.00, 0.10]	0.302	0.071
Basophils percentage, %	0.20[0.10, 0.30]	0.10[0.10, 0.20]	0.482	0.403
NLR	8.86[5.78, 14.04]	11.72[7.88, 18.35]	0.171	0.350
PLR	218.82[160.18, 296.00]	239.28[168.79, 313.77]	0.674	0.660
LMR	5.04[2.21, 5.90]	1.60[1.07, 4.37]	0.008	0.176
SII	1529.98[1037.17, 4119.90]	2428.58[1394.58, 3344.81]	0.443	0.636
CRP, mg/dL	3.71[2.22, 8.13]	3.99[0.90, 11.55]	0.776	0.877
hs-CRP, mg/L	26.26[16.64, 52.36]	12.16[4.84, 46.05]	0.126	0.722

mRS: modified Rankin Scale; MT: mechanism thrombectomy; WBC: white blood cell; NLR: neutrophil-to-lymphocyte ratio: indicating the ratio of neutrophil count to lymphocyte count; LMR: lymphocyte-to-monocyte ratio: indicating the ratio of lymphocyte count to monocyte count; PLR: the platelet-to-lymphocyte ratio: indicating the ratio of platelet count to lymphocyte count; and SII: systemic immune-inflammation index: indicating platelet×neutrophil/lymphocyte; CRP: C-reactive protein; hs-CRP: hyper-sensitive C-reaction protein; *Adjusted for age, OPT, NIHSS at admission, thrombectomy site, pre-MT ASPECTS

### A meta-analysis of the association between inflammatory parameters and prognosis after thrombectomy

**Fig. 1:**
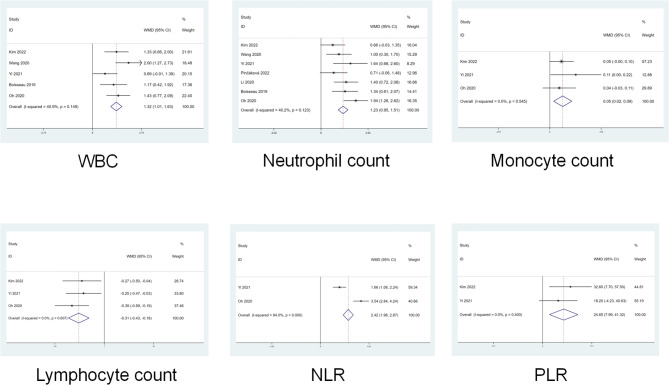
a meta analysis for the association of pre-MT inflammatory indices to 3-month mRS. Compared with patients with 3-month mRS 0–2, WBC count, neutrophil count, monocyte count, NLR and PLR, were higher in patients with 3-month mRS ≥ 3, and the lymphocyte count was lower in this cohort

The screening strategy identified a total of 18 publications for this review (the screening flow chart is detailed in supplemental material Figure S2, and a funnel plot evaluating publication bias is shown in Figure S3) [11–28]. The parameters with means and SD were finally included in the meta-analysis. Compared to patients with 3-month mRS < 3, the pre-MT inflammatory indices, including WBC counts (weighted mean difference, 95%CI, 1.32, 1.01—1.63), neutrophil counts (1.23, 0.95—1.51), monocyte counts (0.05, 0.02—0.09), NLR (2.42, 1.98—2.87) and PLR (24.65, 7.99—41.32), were higher in patients with 3-month mRS ≥ 3, and the lymphocyte counts (−0.31,−0.43 to −0.18) was lower in this cohort (Fig. 1). A summary of the results of the included publications is presented in Table S9 [11–28]. The post-MT inflammatory indices were not available for meta-analysis. Even then, all the results obtained from the involved studies showed that a higher neutrophil counts and NLR, and lower lymphocyte counts are associated with poor prognosis (Table S10) [13, 15–17].

## Discussion

The presented study showed that pre-MT monocyte counts were correlated with the follow-up mRS, however, there was no difference in the pre-MT inflammatory indices between patients who survived and those who died, as well as patients with mRS 0–2 and mRS 3–6 scores at follow-up. We also found that post-MT lymphocyte percentage, monocyte counts, NLR, and LMR were correlated with the follow-up mRS. Post-MT WBC counts, CRP levels and hs-CRP levels were significantly different between patients who survived and those who died. However, there was no difference in the post-MT inflammatory indices between patients with mRS 0–2 and mRS 3–6 scores at follow-up. In addition, ROC analysis showed that post-MT CRP levels can predict death, and post-MT monocyte counts can predict poor outcomes after MT. Hence, post-MT CRP levels and monocyte counts seemed to be more available to predict the prognosis of patients undergoing MT.

A meta-analysis of published studies showed that pre-MT inflammatory indices, including WBC counts, neutrophil counts, monocyte counts, lymphocyte counts, NLR and PLR, were capable of predicting the prognosis of patients treated with MT. In addition, the post-MT inflammatory indices including neutrophil counts, NLR, and lymphocyte counts also appeared to predict the prognosis of patients treated with MT, as demonstrated by the results of previous studies. Hence, the assessment of inflammatory indices may be imperative for predicting the long-term clinical outcomes of patients with MT.

Inflammatory markers have been widely used in the prediction of poor prognosis and complications of ischemic stroke [6, 29]. After an ischemic stroke, several proinflammatory factors, such as brain-derived antigens and damage-associated molecular patterns (DAMPs), enter the body’s circulation from the injured brain region, resulting in intense inflammatory responses that eventually disrupt the blood–brain barrier (BBB) [30]. Simultaneously, circulating neutrophils infiltrate the injured tissue within the first hour after the stroke, releasing proteolytic enzymes, such as arachidonic acid derivatives, superoxide radicals, and matrix metalloproteinase [31]. These enzymes can degrade tight junction proteins and basal lamina type-IV collagen, leading to BBB disruption and tissue damage [32]. Hence, neutrophils can promote an excessive inflammatory response and are considered to be correlated with poor prognosis in stroke patients. The stress caused by stroke during the acute phase activates the hypothalamic-pituitary-adrenal axis, leading to cortisol secretion and excessive sympathetic tone activation, both of which decrease lymphocyte levels [33]. Lymphocytes have a downregulation effect on inflammation; thus, lower lymphocyte levels might indicate milder symptoms and better prognosis [29]. Monocytes participate in inflammatory and prothrombotic pathways by interacting with platelets and endothelial cells in AIS and are also associated with the prognosis of AIS [34]. These immunological cells release various inflammatory factors, further promoting complex inflammatory responses [29]. In theory, the inflammatory response also affects the efficacy of MT after AIS. Excessive inflammatory activation disrupts the BBB and brain tissue, resulting in a high risk of intracranial hemorrhage and malignant brain edema, both of which are strongly associated with the prognosis of AIS patients [7]. 

Although some studies have reported an association between inflammatory indices and the prognosis of AIS patients undergoing MT, the results are diverse. We collected data from our hospital and performed a meta-analysis for this issue in this article. Most of the publications present pre-MT inflammatory indices, including pre-MT WBC counts, neutrophil counts, lymphocyte counts, monocyte counts, NLR, PLR, LMR, and SII, which were strongly associated with prognosis after MT [11–28]. Our pooled results showed that higher pre-MT WBC counts, neutrophil counts, monocyte counts, NLR and PLR, and lower lymphocyte counts were associated with poor long-term outcomes (There were only two studies involved into the analysis of NLR, so the heterogeneity was inevitable. Even so, the involved two study reached the same conclusions that higher pre-MT NLR was associated with poor long-term outcomes). However, our findings do not yield similar results, showing that only monocyte counts was significantly different between patients with mRS 0–2 and mRS ≥ 3 at follow-up after univariate analysis but not after multivariate analysis. Moreover, our study showed no difference in the pre-MT inflammatory indices between patients who survived and those who died. We also performed ROC analysis for the pre-MT parameters that had between-group differences with a *p*-value < 0.1, and no parameter could be used as a predictor of prognosis or death after MT. In theory, pre-MT inflammation is capable of affecting the efficacy of MT; however, the prognosis is more likely to be influenced by a series of factors such as OPT, number of thrombectomy device passes, ASPECTS, and intracranial hemorrhage after reperfusion [35–37]. Many confounding factors affect the predictive power of inflammatory indices. In this respect, pre-MT assessment is ineffective, and thus we considered that pre-MT inflammatory indices might be underpowered to predict prognosis after MT.

In contrast, the post-MT assessment appears to be a promising candidate for the prediction of prognosis after MT. The influencing factors before, during, and after MT may affect the body’s inflammatory status. This multi-collinearity renders the post-MT inflammatory indices powerful for predicting prognosis after MT. As expected, our findings indicated that post-MT lymphocyte percentage, monocyte counts, NLR, and LMR were correlated with the follow-up mRS; post-MT LMR and CRP levels in those who survived were significantly different from those who died when performing univariate analysis, and post-MT WBC counts, CRP levels and hs-CRP levels were significantly different between the two arms when conducting multivariate analysis. Furthermore, univariate analysis showed that post-MT monocyte counts, monocyte percentage and LMR were significantly different between patients with mRS 0–2 and mRS ≥ 3 at follow-up, despite without statistical significance after undergoing multivariate analysis. ROC analysis showed that post-MT CRP levels, with an optimal cutoff value of 4.565, can predict death, and post-MT monocyte counts, with an optimal cutoff value of 0.345, can predict poor outcomes after MT. Previous studies have demonstrated that the post-MT inflammatory status plays an essential role in prognosis after MT [13, 15–17]. They regarded neutrophil counts, lymphocyte counts, and NLR as appropriate prognostic predictors. In brief, the post-MT inflammatory status may be an important factor influencing the prognosis of patients undergoing MT.

This retrospective analytical cohort had several limitations. First, the small sample size may have biased the results toward null hypothesis. Apart from post-MT CRP and monocyte counts, other inflammatory indices such as neutrophil counts, lymphocyte counts, NLR, and LMR might also predict prognosis after MT if the sample size is larger. In addition, different sample sizes were used to assess different markers, which might have influenced the results. We performed a meta-analysis with previous studies to further offset this limitation. Second, because of the retrospective nature of the study, bias in data collection and missing data might have influenced our results. This is a case-control study actually, rather than cross-section study. Whereby, the presentation percentage cannot be used as epidemic data for these populations due to high selection bias. Third, other acknowledged inflammatory markers, such as interleukin (IL)-6, IL-10, and tumor necrosis factor (TNF)-α, were not included in this study because of the limitations of data collection. These indices also appeared to be suitable for the prediction of prognosis after MT and could even be better than the indices investigated in this study; however, their predictive power is still unknown. Further investigations are warranted to address these issues. Fourth, the recruited patients had severe neurological deficits and were comorbid with several complications at admission. Therefore, the mRS scores and death rates were high in this cohort, rendering the between-group sample size unbalanced. In addition, the average follow-up time was 47.5 ± 23.9 days, rather than 30-day or 90-day. This was because we conducted the follow-up evaluation in the outpatient setting, so we could not guarantee the follow-up evaluation performed on time. This limitation might also affect our results. Fifth, other well-known predictive factors for outcome such as collateral status, clot features and penumbra were not investigated in this study. However, we considered inflammatory markers, especially the post-MT indices might had high multi-collinearity with other other predictive factors because they might also affect the body’s immune system.

## Conclusions

The post-MT peripheral inflammatory markers in AIS patients undergoing MT, such as WBC counts, CRP levels and hs-CRP levels were significantly associated with the prognosis of these patients. Post-MT CRP levels can predict death, and monocyte counts can predict poor outcomes after MT, which seemed to be more available to predict the prognosis of patients undergoing MT. Previous studies also indicated that pre-MT inflammatory indices (WBC counts, neutrophil counts, monocyte counts, lymphocyte counts, NLR and PLR) as well as the post-MT inflammatory indices (neutrophil counts, NLR, and lymphocyte counts) were capable of predicting the prognosis of patients treated with MT. Considering the limitations of this study, future prospective studies with a larger sample size and a greater variety of inflammatory markers are necessary to draw more convincing conclusions.

### Electronic supplementary material

Below is the link to the electronic supplementary material.

Supplementary Material 1

## Data Availability

No datasets were generated or analysed during the current study.

## Abbreviations

MT: Mechanical thrombectomy
AIS: acute ischemic stroke
OPT: onset-to-puncture time
ICA: internal carotid artery
MCA: middle cerebral artery
BA: basilar artery
VA: vertebral artery
mRS: modified Rankin Scale
SD: standard deviation
IQR: interquartile range
OR: odds ratio
RR: relative risk
WBC: white blood cells
CRP: C-reactive protein
hs-CRP: hyper-sensitive C-reaction protein
NLR: neutrophil-to-lymphocyte ratio
LMR: lymphocyte-to-monocyte ratio
PLR: platelet-to-lymphocyte ratio
SII: systemic immune-inflammation index
ROC: receiver operating characteristic
+PV: positive predictive value
-PV: negative predictive value
+LR: positive likelihood ratio
-LR: negative likelihood ratio
AUC: area under the ROC curve
DAMPs: damage-associated molecular patterns
BBB: blood–brain barrier
IL: interleukin
