# Supplementary material for: Inflammatory biomarkers may be associated with poor outcomes after mechanical thrombectomy
Source: Thromb J. 2024 Jul 9;22:58. doi: 10.1186/s12959-024-00630-7 (PMC11234743; doi:10.1186/s12959-024-00630-7)
Supplement: Supplementary file 1 — Supplementary Material 1 [file 12959_2024_630_MOESM1_ESM.doc]

**Supplementary Material**

**Table S1. Search strategies**

**Database 1. Pubmed**

**Search date: 2023.3.11**

**Search Strategy:**

#1 exp thrombectomy/

#2 exp embolectomy/

#3 ((bridg* or endovascular) and (therapy or treatment or intervention)) [ti]

#4 (thrombectomy or embolectomy or endovascular) [ti]

#5 (revascular*) [ti]

#6 #1 or #2 or #3 or #4 or #5

#7 exp brain ischemia/

#8 exp stroke/

#9 exp intracranial embolism and thrombosis/

#10 ((cerebrovascular or cerebral arter*) and (accident or event* or disorder* or disease*)) [ti]

#11 ((intracranial or carotid arter* or brain or cerebr*) and (ischemi* or embolism or thrombosis or obstruct* or occlus* or block* or infarct* or clot* or arteriosclerosis)) [ti]

#12 (stroke or AIS or apople*) [ti]

#13 (large vessel occlusion* or large arter* occlusion*) [ti]

#14 #7 or #8 or #9 or #10 or #11 or #12 or #13

#15 exp inflammation

#16 (inflammat* or immun*) [ti]

#17 (neutrophil or lymphocyte or monocyte or C-reaction protein or leukocyte or NLR or LMR or PLR or white blood cell) [ti]

#18 (biomarker or parameter or indices) [ti]

#19 #15 or #16 or #17 or #18

#20 #6 and #14 and #19

**Database 2. Embase**

**Search date: 2023.3.7**

**Search Strategy:**

1 exp *thrombectomy/

2 exp *embolectomy/

3 ((bridg* or endovascular) and (therapy or treatment or intervention)).ti.

4 (thrombectomy or embolectomy or endovascular).ti.

5 (revascular*).ti.

6 or 1-5

7 exp *brain ischemia/

8 exp *stroke/

9 *exp intracranial embolism and thrombosis/

10 ((cerebrovascular or cerebral arter*) and (accident or event* or disorder* or disease*)).ti.

11 ((intracranial or carotid arter* or brain or cerebr*) and (isch*emi* or embolism or thrombosis or obstruct* or occlus* or block* or infarct* or clot* or arteriosclerosis)).ti.

12 (stroke or AIS or apople*).ti.

13 (large vessel occlusion* or large arter* occlusion*).ti.

14 or 7-13

15 exp *inflammation

16 (inflammat* or immun*).ti.

17 (neutrophil or lymphocyte or monocyte or C-reaction protein or leukocyte or NLR or LMR or PLR or white blood cell).ti.

18 (biomarker or parameter or indices).ti.

19 or 15-18

20 6 and 14 and 19

21 animal.mh

22 20 and 21

**Database 3. ClinicalTrials. gov**

**Search date: 2023.3.7**

**Search Strategy:**

Thrombectomy AND Cerebrovascular Disorders [DISEASE] AND inflammation

**Table S2.** Differences of baseline characteristics between survivors and deaths after MT

| Characteristics | Survivors | Deaths | p-value |
| --- | --- | --- | --- |
| Num.of patients | 47 | 23 | - |
| Demographics |  |  |  |
| Male, num.(%) | 34 (72.3) | 18 (78.3) | 0.773 |
| Age, years | 64.23±12.03 | 63.35±11.15 | 0.768 |
| History, num.(%) |  |  |  |
| Hypertension | 30 (63.8) | 15 (65.2) | 1.000 |
| Diabetes | 12 (25.5) | 6 (26.1) | 1.000 |
| Atrial fibrillation | 14 (29.8) | 8 (34.8) | 0.785 |
| Brain infarction | 7 (14.9) | 8 (34.8) | 0.070 |
| Smoke | 21 (44.7) | 10 (43.5) | 1.000 |
| Drink | 15 (31.9) | 11 (47.8) | 0.292 |
| Thrombectomy site, num.(%) |  |  |  |
| Internal carotid artery | 12 (25.5) | 10 (43.5) | 0.172 |
| Middle cerebral artery | 25 (53.2) | 5 (21.7) | 0.020 |
| Basal artery | 9 (19.1) | 8 (34.8) | 0.234 |
| Vertebral artery | 1 (2.1) | 0 (0.0) | 1.000 |
| Clinical presentations |  |  |  |
| OPT, hours | 6.50±3.48 | 6.47±2.38 | 0.971 |
| NIHSS score | 14.77±6.78 | 21.00±6.63 | 0.001 |
| Pre-MT ASPECTS | 9.0 (9.0, 10.0) | 9.0 (7.0, 10.0) | 0.016 |
| Post-MT ASPECTS | 7.0 (6.0, 8.0) | 3.0 (2.0, 5.0) | <0.001 |
| OPT, onset-to-puncture time; NIHSS, National Institutes of Health Stroke Scale; ASPECTS, Alberta Stroke Program Early CT Score; MT, mechanism thrombectomy. | | | |

**Table S3.** Differences of baseline characteristics between patients with the follow-up mRS 0-2 and ≥3 after MT

| Characteristics | mRS 0-2 | mRS≥3 | p-value |
| --- | --- | --- | --- |
| Num.of patients | 15 | 55 |  |
| Demographics |  |  |  |
| Male, num.(%) | 10 (66.7) | 42 (76.4) | 0.510 |
| Age, years | 58.13±10.41 | 65.53±11.58 | 0.029 |
| History, num.(%) |  |  |  |
| Hypertension | 10 (66.7) | 35 (63.6) | 1.000 |
| Diabetes | 4 (26.7) | 14 (25.5) | 1.000 |
| Atrial fibrillation | 4 (26.7) | 18 (32.7) | 0.761 |
| Brain infarction | 3 (20.0) | 12 (21.8) | 1.000 |
| Smoke | 8 (53.3) | 23 (41.8) | 0.560 |
| Drink | 6 (40.0) | 20 (36.4) | 1.000 |
| Thrombectomy site, num.(%) |  |  |  |
| Internal carotid artery | 3 (20.0) | 19 (34.5) | 0.359 |
| Middle cerebral artery | 7 (46.7) | 23 (41.8) | 0.775 |
| Basal artery | 5 (33.3) | 12 (21.8) | 0.497 |
| Vertebral artery | 0 (0.0) | 1 (1.8) | 1.000 |
| Clinical presentations |  |  |  |
| OPT, hours | 7.93±4.69 | 6.10±2.49 | 0.044 |
| NIHSS score | 13.73±7.29 | 17.65±7.13 | 0.065 |
| Pre-MT ASPECTS | 10.0 (9.0, 10.0) | 9.0 (8.0, 10.0) | 0.105 |
| Post-MT ASPECTS | 7.0 (6.0, 8.0) | 6.0 (3.0, 8.0) | 0.035 |
| mRS, modified Rankin Scale; MT, mechanism thrombectomy; OPT, onset-to-puncture time; NIHSS, National Institutes of Health Stroke Scale; ASPECTS, Alberta Stroke Program Early CT Score. | | | |

**Table S4.** Association between pre-MT parameters and baseline NIHSS scores.

| Parameter-baseline NIHSS | p-value | Pearson |
| --- | --- | --- |
| WBC count | 0.576 | 0.078 |
| Neutrophil percentage | 0.784 | 0.038 |
| Lymphocyte percentage | 0.620 | -0.069 |
| Monocyte percentage | 0.296 | 0.145 |
| Eosinophils percentage | 0.660 | -0.061 |
| Basophils percentage | 0.296 | -0.145 |
| Neutrophil count | 0.688 | 0.056 |
| Monocyte count | 0.149 | 0.199 |
| Lymphocyte count | 0.826 | 0.031 |
| NLR | 0.864 | 0.024 |
| PLR | 0.853 | -0.026 |
| LMR | 0.194 | -0.179 |
| SII | 0.981 | 0.003 |
| NIHSS, National Institutes of Health Stroke Scale; MT, mechanism thrombectomy; WBC, white blood cell; NLR: neutrophil-to-lymphocyte ratio, indicating the ratio of neutrophil count to lymphocyte count; LMR: lymphocyte-to-monocyte ratio, indicating the ratio of lymphocyte count to monocyte count; PLR: the platelet-to-lymphocyte ratio, indicating the ratio of platelet count to lymphocyte count; and SII: systemic immune-inflammation index, indicating platelet×neutrophil/lymphocyte. | | |

**Table S5.** Association between pre-MT parameters and the follow-up mRS scores.

| Parameter | p-value | Spearman |
| --- | --- | --- |
| WBC count | 0.285 | 0.148 |
| Neutrophil percentage | 0.571 | -0.079 |
| Lymphocyte percentage | 0.885 | 0.020 |
| Monocyte percentage | 0.246 | 0.161 |
| Eosinophils percentage | 0.557 | 0.082 |
| Basophils percentage | 0.654 | -0.062 |
| Neutrophil count | 0.658 | 0.062 |
| Monocyte count | 0.021 | 0.314 |
| Lymphocyte count | 0.451 | 0.105 |
| NLR | 0.765 | -0.042 |
| PLR | 0.276 | -0.151 |
| LMR | 0.211 | -0.173 |
| SII | 0.675 | -0.058 |
| mRS, modified Rankin Scale; WBC, white blood cell; NLR: neutrophil-to-lymphocyte ratio, indicating the ratio of neutrophil count to lymphocyte count; LMR: lymphocyte-to-monocyte ratio, indicating the ratio of lymphocyte count to monocyte count; PLR: the platelet-to-lymphocyte ratio, indicating the ratio of platelet count to lymphocyte count; and SII: systemic immune-inflammation index, indicating platelet×neutrophil/lymphocyte. | | |

**Table S6.** ROC analysis for inflammatory markers to predict death after MT

| Biomarkers | AUC | p-value | 95%CI |
| --- | --- | --- | --- |
| Pre-MT |  |  |  |
| WBC count | 0.660 | 0.066 | 0.493-0.826 |
| Monocyte count | 0.661 | 0.063 | 0.512-0.811 |
| Post-MT |  |  |  |
| WBC count | 0.640 | 0.062 | 0.506-0.773 |
| Monocyte count | 0.641 | 0.060 | 0.500-0.781 |
| Lymphocyte percentage | 0.356 | 0.055 | 0.212-0.500 |
| NLR | 0.636 | 0.068 | 0.492-0.781 |
| LMR | 0.332 | 0.025 | 0.200-0.464 |
| SII | 0.626 | 0.091 | 0.484-0.769 |
| CRP | 0.737 | 0.005 | 0.587-0.887 |
| hs-CRP | 0.710 | 0.061 | 0.501-0.920 |
| Parameters with p<0.1 were included in ROC analysis. MT, mechanism thrombectomy; WBC, white blood cell; NLR: neutrophil-to-lymphocyte ratio, indicating the ratio of neutrophil count to lymphocyte count; LMR: lymphocyte-to-monocyte ratio, indicating the ratio of lymphocyte count to monocyte count; PLR: the platelet-to-lymphocyte ratio, indicating the ratio of platelet count to lymphocyte count; and SII: systemic immune-inflammation index, indicating platelet×neutrophil/lymphocyte; CRP, C-reactive protein; hs-CRP, hyper-sensitive C-reaction protein. | | | |

**Table S7.** ROC analysis for inflammatory markers to predict poor outcomes after MT

| Biomarkers | AUC | p-value | 95%CI |
| --- | --- | --- | --- |
| Pre-MT |  |  |  |
| WBC count | 0.400 | 0.327 | 0.221-0.579 |
| Monocyte count | 0.689 | 0.065 | 0.504-0.874 |
| Post-MT |  |  |  |
| Monocyte count | 0.704 | 0.017 | 0.575-0.833 |
| Monocyte percentage | 0.683 | 0.032 | 0.545-0.820 |
| LMR | 0.273 | 0.008 | 0.146-0.400 |
| Parameters with p<0.1 were included in ROC analysis. MT, mechanism thrombectomy; WBC, white blood cell; LMR: lymphocyte-to-monocyte ratio, indicating the ratio of lymphocyte count to monocyte count. | | | |

**Table S8.** Association between post-MT parameters and the follow-up mRS scores.

| Parameter | p-value | Spearman |
| --- | --- | --- |
| WBC count | 0.058 | 0.233 |
| Neutrophil percentage | 0.234 | 0.147 |
| Lymphocyte percentage | 0.019 | -0.287 |
| Monocyte percentage | 0.139 | 0.183 |
| Eosinophils percentage | 0.378 | -0.109 |
| Basophils percentage | 0.386 | -0.108 |
| Neutrophil count | 0.063 | 0.228 |
| Monocyte count | 0.030 | 0.266 |
| Lymphocyte count | 0.192 | -0.161 |
| NLR | 0.026 | 0.272 |
| PLR | 0.370 | 0.111 |
| LMR | 0.009 | -0.317 |
| SII | 0.087 | 0.211 |
| CRP count | 0.069 | 0.256 |
| hs-CRP count | 0.919 | -0.017 |
| mRS, modified Rankin Scale; WBC, white blood cell; NLR: neutrophil-to-lymphocyte ratio, indicating the ratio of neutrophil count to lymphocyte count; LMR: lymphocyte-to-monocyte ratio, indicating the ratio of lymphocyte count to monocyte count; PLR: the platelet-to-lymphocyte ratio, indicating the ratio of platelet count to lymphocyte count; and SII: systemic immune-inflammation index, indicating platelet×neutrophil/lymphocyte; CRP, C-reactive protein; hs-CRP, hyper-sensitive C-reaction protein. | | |

**Table S9.** The association of pre-MT inflammatory indices with the prognosis after MT

| Biomarkers | Articles | 3-month mRS 0-2 (Number of patients) | 3-month mRS≥3 (Number of patients) | p-value |
| --- | --- | --- | --- | --- |
| WBC count, 10^9^/L | Kim, 2022 | 8.35±3.29 (240) | 9.68±3.73 (192) | 0.104 |
|  | Wang, 2020 | 9.00±2.70 (140) | 11.00±3.80 (169) | <0.001 |
|  | Chen, 2021 | 7.80[6.70, 10.60] (86) | 8.50[7.00, 10.80] (171) | 0.107 |
|  | Yi, 2021 | 8.92±3.06 (245) | 9.61±4.14 (195) | 0.202 |
|  | Pinčáková, 2022 | 8.32[6.99, 9.89] (78) | 8.24[6.82, 10.90] (101) | 0.599 |
|  | Boisseau, 2019 | 9.77±2.92 (134) | 10.94±3.86 (180) | 0.004 |
|  | Oh, 2020 | 8.88±2.80 (212) | 10.31±3.99 (199) | 0.022 |
|  | Ozgen, 2020 | 9.50[4.30, 27.40] (58) | 10.20[6.40, 20.70] (92) | 0.059 |
| Neutrophil count, 10^9^/L | Kim, 2022 | 6.77±3.38 (240) | 7.43±3.81 (192) | 0.032 |
|  | Li, 2022 | 6.60[4.20, 8.80] (115) | 6.50[4.70, 9.00] (143) | 0.721 |
|  | Feng, 2022 | 6.01[4.90, 8.28] (100) | 7.35[4.57, 9.80] (70) | 0.184 |
|  | Wang, 2020 | 8.00±2.60 (140) | 9.00±3.70 (169) | <0.001 |
|  | Yi, 2021 | 6.47±3.79 (245) | 8.11±5.92 (195) | 0.033 |
|  | Chen, 2021 | 5.90[4.40, 8.10] (86) | 6.60[5.10, 9.20] (171) | 0.002 |
|  | Pinčáková, 2022 | 5.88±2.08 (78) | 6.59±3.13 (101) | 0.076 |
|  | Li, 2020 | 5.30±2.10 (101) | 6.70±3.00 (120) | <0.001 |
|  | Ferro, 2021 | 7.12[4.95, 9.16] (178) | 6.03[4.17, 7.65] (147) | - |
|  | Semerano, 2019 | 5.30[4.00, 7.00] (191) | 6.00[4.40, 8.40] (242) | 0.005 |
|  | Boisseau, 2019 | 7.30±2.90 (134) | 8.64±3.66 (180) | <0.001 |
|  | Li, 2021 | 6.50[5.86, 7.20] (148) | 6.92[6.24, 7.91] (138) | <0.001 |
|  | Oh, 2020 | 6.07±2.63 (212) | 8.01±4.19 (199) | 0.010 |
|  | Ozgen, 2020 | 6.80[1.80, 23.70] (58) | 8.50[3.20, 17.30] (92) | 0.003 |
| Lymphocyte count, 10^9^/L | Kim, 2022 | 1.99±1.03 (240) | 1.72±1.37 (192) | 0.030 |
|  | Li, 2022 | 1.30[0.90, 1.80] (115) | 1.20[0.70, 1.90] (143) | 0.297 |
|  | Feng, 2022 | 1.43[1.00, 1.90] (100) | 1.26[0.74, 1.73] (70) | 0.080 |
|  | Yi, 2021 | 1.97±1.21 (245) | 1.72±1.09 (195) | 0.107 |
|  | Chen, 2021 | 1.40[1.00, 2.00] (86) | 1.10[0.70, 1.70] (171) | 0.000 |
|  | Pinčáková, 2022 | 1.81[1.31, 2.44] (78) | 1.47[1.03, 2.07] (101) | 0.009 |
|  | Ferro, 2021 | 0.99[0.68, 1.41] (178) | 1.31[0.96, 1.92] (147) | - |
|  | Semerano, 2019 | 1.80[1.20, 2.50] (191) | 1.60[1.10, 2.30] (242) | 0.235 |
|  | Boisseau, 2019 | 1.49[1.12, 2.02] (134) | 1.34[0.91, 1.72] (180) | 0.014 |
|  | Li, 2021 | 1.91[1.43, 2.38] (148) | 1.28[0.91, 1.84] (138) | <0.001 |
|  | Oh, 2020 | 2.05±1.01 (212) | 1.66±1.10 (199) | 0.008 |
|  | Ozgen, 2020 | 1.90[1.00, 4.60] (58) | 1.30[0.50, 4.80] (92) | <0.001 |
| Monocyte count, 10^9^/L | Kim, 2022 | 0.56±0.24 (240) | 0.61±0.29 (192) | 0.251 |
|  | Li, 2022 | 0.40[0.20, 0.60] (115) | 0.40[0.20, 0.50] (143) | 0.195 |
|  | Yi, 2021 | 0.71±0.52 (245) | 0.82±0.61 (195) | 0.664 |
|  | Pinčáková, 2022 | 0.43[0.35, 0.51] (78) | 0.45[0.33, 0.60] (101) | 0.134 |
|  | Li, 2021 | 0.45[0.33, 0.58] (148) | 0.50[0.38, 0.60] (138) | 0.056 |
|  | Oh, 2020 | 0.56±0.31 (212) | 0.60±0.41 (199) | 0.924 |
|  | Ozgen, 2020 | 0.50[0.20, 8.20] (58) | 0.50[0.10, 1.30] (92) | 0.935 |
| NLR | Li, 2022 | 4.90[2.50, 8.90] (115) | 5.70[2.40, 12.20] (143) | 0.315 |
|  | Ma, 2022 | 4.85[4.96] (331) | 6.57[7.21] (465) | <0.001 |
|  | Bartt, 2022 | 4.00[2.00, 6.00] (223) | 6.00[3.00, 10.00] (197) | 0.010 |
|  | Aly, 2020 | 2.60[1.70, 5.10] (63) | 3.50[1.90, 5.10] (79) | 0.465 |
|  | Feng, 2022 | 4.17[2.65, 6.99] (100) | 5.85[2.63, 10.67] (70) | 0.044 |
|  | Yi, 2021 | 3.31±2.79 (245) | 4.97±3.27 (195) | 0.019 |
|  | Chen, 2021 | 4.10[2.30, 6.80] (86) | 5.90[3.40, 11.30] (171) | 0.000 |
|  | Pinčáková, 2022 | 3.02[1.92, 4.42] (78) | 3.78[2.25, 5.65] (101) | 0.077 |
|  | Ferro, 2021 | 7.71[4.36, 11.70] (178) | 4.01[2.51, 8.39] (147) | - |
|  | Semerano, 2019 | 2.70[1.90, 4.60] (191) | 3.50[2.10, 6.70] (242) | 0.004 |
|  | Boisseau, 2019 | 4.70[2.80, 7.30] (134) | 6.60[3.70, 10.10] (180) | <0.001 |
|  | Li, 2021 | 3.44[2.63, 4.63] (148) | 5.28[3.63, 8.02] (138) | <0.001 |
|  | Oh, 2020 | 3.78±2.86 (212) | 7.32±4.18 (199) | <0.001 |
|  | Shi, 2021 | 6.29[3.58, 9.06] (69) | 5.35[3.33, 10.04] (58) | 0.742 |
|  | Ozgen, 2020 | 3.50[0.40, 21.60] (58) | 6.90[0.70, 24.70] (92) | <0.001 |
| PLR | Kim, 2022 | 135.0±120.3 (240) | 167.6±139.3 (192) | <0.001 |
|  | Ma, 2022 | 153.90[113.05] (331) | 168.89[140.10] (465) | 0.002 |
|  | Yi, 2021 | 119.2±108.5 (245) | 137.4±127.2 (195) | 0.040 |
|  | Pinčáková, 2022 | 126.99[91.19, 164.37] (78) | 123.73[95.27, 203.70] (101) | 0.629 |
|  | Ferro, 2021 | 212.00[127.00, 306.00] (178) | 153.00[99.00, 239.00] (147) | - |
|  | Ozgen, 2020 | 124.00[11.20, 229.10] (58) | 161.00[62.10, 541.40] (92) | <0.001 |
| LMR | Ma, 2022 | MLR 0.28[0.17] (331) | 0.32[0.25] (465) | <0.001 |
|  | Yi, 2021 | 2.81±2.03 (245) | 1.92±1.72 (195) | 0.027 |
|  | Pinčáková, 2022 | 4.20[3.16, 5.72] (78) | 3.50[2.21, 5.06] (101) | 0.008 |
| SII | Yi, 2021 | 741±674 (245) | 1044±897 (195) | 0.004 |
| -: data not provided. NLR: neutrophil-to-lymphocyte ratio, indicating the ratio of neutrophil count to lymphocyte count; LMR: lymphocyte-to-monocyte ratio, indicating the ratio of lymphocyte count to monocyte count; PLR: the platelet-to-lymphocyte ratio, indicating the ratio of platelet count to lymphocyte count; and SII: systemic immune-inflammation index, indicating platelet×neutrophil/lymphocyte. | | | | |

| Biomarkers | Articles | 3-month mRS 0-2 (Number of patients) | 3-month mRS≥3 (Number of patients) | p-value |
| --- | --- | --- | --- | --- |
| Neutrophil count, 10^9^/L | Li, 2022 | 7.50[6.00, 9.60] (115) | 9.70[8.40, 11.90] (143) | <0.001 |
|  | Feng, 2022 | 7.31[5.45, 8.96] (100) | 8.57[6.81, 11.39] (70) | 0.001 |
| Lymphocyte count, 10^9^/L | Li, 2022 | 1.20[0.90, 1.70] (115) | 0.80[0.60, 1.00] (143) | <0.001 |
|  | Feng, 2022 | 1.19[0.85, 1.47] (100) | 0.80[0.48, 1.23] (70) | <0.001 |
| Monocyte count, 10^9^/L | Li, 2022 | 0.60[0.30, 0.70] (115) | 0.50[0.10, 0.80] (143) | 0.191 |
| NLR | Li, 2022 | 6.70[4.10, 8.60] (115) | 11.70[10.00, 15.50] (143) | <0.001 |
|  | Bartt, 2022 | 6.00[3.50, 8.00] (223) | 7.00[5.00, 10.00] (197) | <0.01 |
|  | Aly, 2020 | 4.00[2.80, 5.80] (63) | 6.10[4.00, 10.90] (79) | <0.001 |
|  | Feng, 2022 | 6.82[4.21, 9.45] (100) | 10.87[6.39, 19.00] (70) | <0.001 |
| NLR: neutrophil-to-lymphocyte ratio, indicating the ratio of neutrophil count to lymphocyte count. | | | | |

**Table S10.** The association of post-MT inflammatory indices with the prognosis after MT

**
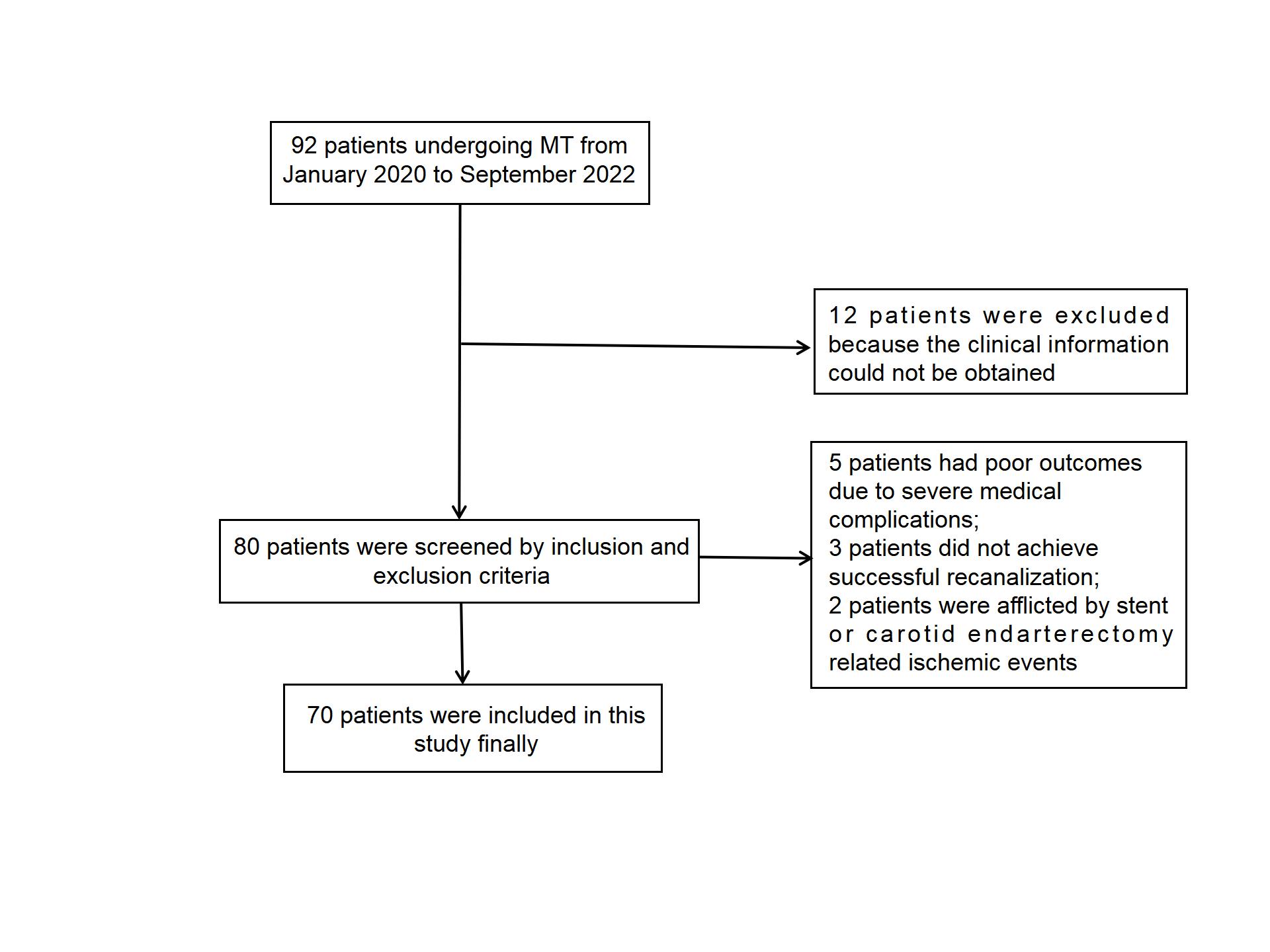
**

**Figure S1.** Flowchart for patient selection


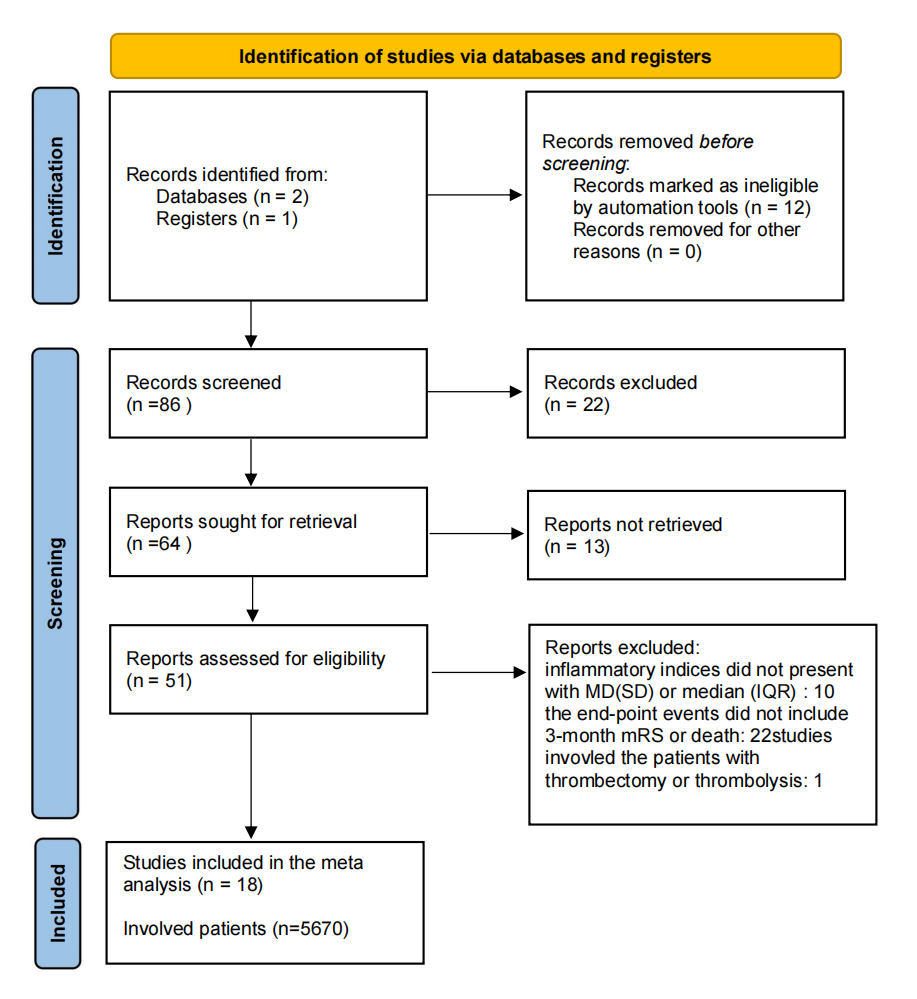


**Figure S2.** Search strategy for the systematic review.


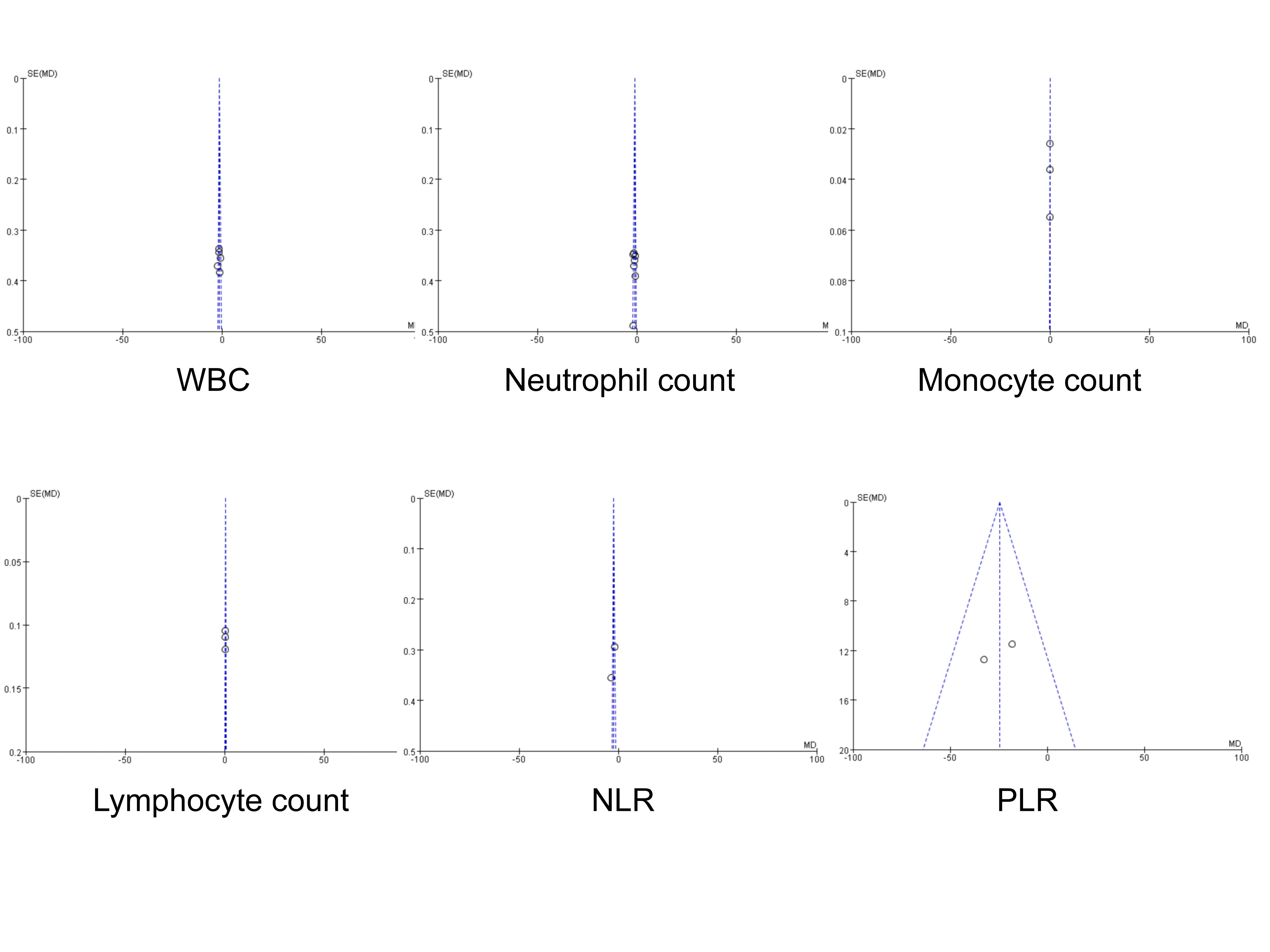


**Figure S3.** Funnel plot for the publication bias. As the figure presents, the graph is symmetrical which demonstrates that the publication bias of included studies is low.
